# Diagnostic Performance of 1→3-β-d-Glucan in Neonatal and Pediatric Patients with Candidemia

**DOI:** 10.3390/ijms12095871

**Published:** 2011-09-14

**Authors:** Maria Teresa Montagna, Caterina Coretti, Grazia Lovero, Osvalda De Giglio, Osvaldo Montagna, Nicola Laforgia, Nicola Santoro, Giuseppina Caggiano

**Affiliations:** 1 Department of Biomedical Science and Human Oncology—Hygiene Section, University of Bari, “Aldo Moro”, Piazza G. Cesare 11, 70124 Bari, Italy; E-Mails: catiacoretti@yahoo.it (C.C.); raz.ro@tiscali.it (G.L.); osvaldadegiglio@tiscali.it (O.D.G.); caggianog@igiene.uniba.it (G.C.); 2 Department of Gynecology, Obstetrics and Neonatology—Neonatology and Neonatology Intensive Care Section, University of Bari, “Aldo Moro”, Piazza G. Cesare 11, 70124 Bari, Italy; E-Mails: osvaldo.montagna@tin.it (O.M.); n.laforgia@neonatologia.uniba.it (N.L.); 3 U.O. Pediatrics “F.Vecchio”, A.O.U. Policlinico Consorziale, Piazza G. Cesare 11, 70124 Bari, Italy; E-Mail: n.santoro@bioetaev.uniba.it

**Keywords:** candidemia, 1→3-β-d-glucan, mannan antigen, pediatric patients

## Abstract

Fungal sepsis is one of the major problems in neonatal and pediatric care unit settings. The availability of new diagnostic techniques could allow medical practitioners to rapidly identify septic patients and to improve their outcome. The aim of this study was to evaluate the performance of the 1→3-β-d-glucan (BDG), individually and in comparison with the *Candida* mannan (CM) antigen, in ten preterm infants and five onco-haematological pediatric patients with *Candida* bloodstream infections already proven by positive culture. The serum levels of BDG were >80 pg/mL on the same day as a positive blood culture in all examined patients, while CM antigen was negative in the patients with *C. parapsilosis* fungemia and in one further case due to *C. albicans*. These results suggest that a regular monitoring of serum circulating antigens (*i.e*., 1→3-β-d-glucan) combined with other microbiological and clinical information, may allow earlier and accurate diagnosis. However, further studies are necessary to confirm its usefulness in routine clinical practice.

## 1. Introduction

Despite extensive research and development in the diagnostic and therapeutic fields, *Candida* bloodstream infections (BSI) remain an important cause of morbidity and mortality in neonatal intensive care units (NICU) and in high risk patients, particularly in immunocompromised ones. An early diagnosis of invasive fungal disease (IFD) is essential in this population, but the infection is difficult to identify because signs and symptoms are often minimal and similar to those of various other non-infectious processes. In addition, the diagnosis of candidemia is still primarily limited to standard blood cultures, but it is known that traditional methods of microbiological cultures are often insensitive or require several days to yield fungi and test their susceptibility to drugs [1,2]. Another important factor that can influence the reliability of culture methods is previous prophylaxis or empirical treatments with antifungal drugs. For these reasons, other laboratory tools were studied. Among these, serological tests are difficult to interpret because the circulating antibodies to *Candida* spp. may occur in healthy subjects as a result of commensal colonization of mucosal surfaces. Furthermore, their production in the immunocompromised patients varies according to immune status [3]. In these complex scenarios, newer diagnostic methods, including biochemical markers, the polymerase chain reaction and circulating antigen assays, were made available but are not commonly employed and still require standardization and further evaluation.

The detection of *Candida* mannan antigen (CM) has shown encouraging results in terms of sensitivity (94.4%) and specificity (94.2%) in neonatal patients with candidemia [4], but its levels in blood can be low and the transient nature of antigenaemia requires repetitive sampling. Another serum marker recently studied in patients with deep mycoses is 1→3-β-d-glucan (BDG) [5,6], which has been included among the relevant diagnostic criteria by the European Organization for Research and Treatment of Cancer/Mycoses Study Group (EORTC/MSG) [7]. BDG is a component of the cell wall of a wide variety of fungi except for zigomycetes and, to a lesser extent, *Cryptococcus* spp. [8,9]. However, only a few reports specifically describe the clinical relevance of BDG in preterm infants or onco-haematological pediatrics with candidemia [10]. Previous data regarding the pediatric population derive from a study carried out on healthy children showing BDG levels higher than those reported in adults, with a small number of false-positive results [11].

The aim of this study was to evaluate the performance of the BDG test, individually and in comparison with CM antigen, in neonatal and pediatric patients with a *Candida* BSI.

## 2. Material and Methods

We examined fifteen children with *Candida* BSI already proven by positive culture: ten preterm infants (gestational age < 37 weeks) and five onco-haematological children, admitted to the Neonatal Intensive Care Unit and to the Haematology Unit of a large University Hospital in Southern Italy.

In all patients, serum BDG and CM antigens were tested on the same day as the positive blood culture and repeated on a sample drawn 24 h later.

Blood cultures were performed using the lyses centrifugation system (Isolator^®^, DuPont Co., Wilmington, Delaware) and were cultured on Sabouraud agar plates with gentamicin-chloramphenicol (Becton-Dickinson, Heidelberg, Germany), incubated at 36 ± 1 °C and examined daily.

BDG detection was performed by colorimetric assay, Fungitell (Associates of Cape Cod Inc., E. Falmouth, MA, USA), and each serum was tested in triplicate. Serum that was haemolysed, lipemic or visually icteric or turbid was not suitable for the assays. BDG levels ≥80 pg/mL were considered as positive, ranging from 60 to 79 pg/mL as indeterminate, <60 pg/mL as negative.

CM antigen was assayed using a commercial sandwich enzyme-linked immunoassay, Platelia *Candida* Ag (BioRad, Marnes La Coquette, France). Antigen values ≥0.5 ng/mL were considered as positive, ranging from 0.25 to 0.49 ng/mL as intermediate and <0.25 ng/mL as negative.

Both tests were performed according to the manufacturer's instructions.

As negative controls, 15 hospitalized patients (10 preterm infants, and 5 onco-haematological children) without any clinical evidence of fungal infection (*i.e.*, fever, increased C-reactive protein levels) and with negative blood culture was tested for BDG and CM antigens.

## 3. Results

The demographic/clinical characteristics of patients with candidemia and results of antigens research are summarized in Table 1.

Ten out of the 15 patients were pre-term infants: two Extremely Low Birth Weight (ELBW, <1000 g), five Very Low Birth Weight (VLBW, between 1001 and 1500 g) and three Low Birth Weight (LBW, between 1501 and 2500 g). Overall, six patients developed a BSI caused by *Candida albicans*, three by *Candida parapsilosis* and one by *Candida glabrata*.

On the same day as the positive blood culture, the BDG values were >80 pg/mL in all ten neonates with *Candida* BSI, while the CM antigen values were >0.5 ng/mL only in seven infants (six with *C. albicans* and one with *C. glabrata* BSI). The remaining three infants with *C. parapsilosis* BSI were negative.

Regarding the onco-haematological pediatric patients (three affected by acute lymphoid leukaemia, one by acute myeloid leukaemia and one by non-Hodgkin’s lymphoma), all five children had BDG >80 pg/mL on the same day as the positive blood culture, while the CM antigen resulted positive only in two children (one with *C. albicans* and one with *Candida lusitaniae* fungemia). The remaining three children (two with *C. parapsilosis* and one *C. albicans* BSI) were negative.

These results were confirmed in all the samples drawn 24 h after the first one.

The BDG and *Candida* mannan tests were always negative in all control patients.

## 4. Discussion

The aim of this study is to evaluate the presence of circulating antigens (1→3-β-d-glucan and *Candida* mannan) in the serum of neonatal and pediatric patients with BSI on the same day as the notification of yeasts in their blood culture. Although the number of enrolled patients was low, we consider it interesting to underline the constant correspondence of the BDG antigen test results in all fifteen children with the timing of the positive blood culture, even when the mannan antigen resulted negative. In fact, in all our patients with *C. parapsilosis* BSI, mannan antigen was negative according to literature data that report a very low sensitivity of Platelia *Candida* antigen test in patients with *C. parapsilosis* infection, probably because of the lower amount of mannan released by this species [4,12]. The fact that this assay was negative in each patient with *C. parapsilosis* candidemia suggests that it could not be a useful surrogate marker in this population. This is a real problem, because *C. parapsilosis* representing the second most common species isolated from the pediatric population [13].

However, even considering the greater sensitivity of the Platelia *Candida* antigen test for infections caused by *C. albicans* [14], this data could not be confirmed in any of our patients with *C. albicans* BSIs. Most notably, one out of the five onco-haematological pediatric patients was severely immunocompromised and had a long complicated course of the disease in rapid progression with severe complications but favorable resolution of *C. albicans* BSI. In this patient, the BDG test was positive on the same day as the blood culture, but a negative mannan antigen test was observed in both serum samples tested in two consecutive days under study. For these reasons, taking into account also the low sensitivity of blood cultures for *Candida* detection, especially in neonatal patients [3], and the rapid clearance of mannan antigen from the blood [15], a positive BDG test could raise the suspicion of fungal disease even when the mannan antigen test and/or blood cultures are negative.

Our data are in agreement with Mularoni *et al*. [10] who reported high plasmatic levels of BDG (>523 pg/mL) in serum of four children with documented IFD. Interestingly, in a retrospective study of 27 patients with candidemia (three subjects were pediatric patients), Alam *et al*. [16] evaluated the diagnostic value of BDG individually and in comparison with other disease markers (*Candida* mannan and anti-mannan antibodies), suggesting that the combination of two or more diagnostic assays could help to enhance the sensitivity for diagnosis of candidemia.

In the light of the constant positivity of BDG antigen test in our patients with fungemia, it should be considered that the glucan is ubiquitous in the environment, so some medical sources of BDG can lead to a false positive assay result in the absence of IFI: dialysis membranes and filters made from cellulose, cotton gauze employed during surgery, specific fractionated blood products such as serum albumin and immunoglobulins are reported to contain BDG. Moreover, there are other potential reasons for false positive BDG reactions: exposure to antitumor polysaccharides such as lentinam, polysaccharide K and schizophyllan which are derived from different species of mushrooms, and treatment with certain parenteral antimicrobials [17–19]. We considered these possibilities in the course of the study, and the interpretation of our results was made in view of all these circumstances. The clinical setting of our patients did not appear to be significant and consequently, we can rule out the possibility of such interferences.

In a recent study Mokaddas *et al*. [20] have reported that some pediatric patients colonized with *Candida* spp. had high levels of BDG. We are able to exclude also this possible interference, because in our hospital a weekly microbiological surveillance is carried out in various anatomic sites (*i.e.*, nose, oropharynx, intestine, vagina) of patients at high risk for fungal disease. No fungal colonization was detected in all studied patients.

## 5. Conclusions

We are aware that the this study presents some limitations (*i.e.*, the lack of constant monitoring of BDG levels), but we aimed to evaluate the presence of BDG on the same day of candidemia to establish the role of this assay in the diagnosis of this disease in pediatric population. We think the research of BDG cannot entirely replace the standard diagnostic approaches, but a regular monitoring of this circulating antigens over time, combined with other microbiological and clinical information, may allow earlier and accurate diagnosis in this setting patients and minimize the inappropriate use of drugs. In this context, the goal of this preliminary study is to provide a platform for mycologist scientists to promote and discuss the design of future trials focused to elucidate this issue.

## Figures and Tables

**Table 1:** Clinical features, culture data and values of 1→3-β-d-glucan (BDG) and *Candida* mannan (CM) antigen for 15 patients, timing of documented candidemia.

Patient no.	Age/Gender	Underlying condition	Isolates	BDG (pg/mL)	CM (ng/mL)
1	25 days/M	ELBW (825 g)	*C. albicans*	>523	1.70
2	75 days/M	ELBW (535 g)	*C. parapsilosis*	>523	<0.25
3	25 days/M	VLBW (1065 g)	*C. albicans*	276	1.88
4	20 days/M	VLBW (1010 g)	*C. albicans*	312	1.60
5	103 days/M	VLBW (1145 g)	*C. albicans*	>523	1.53
6	9 days/F	VLBW (1200 g)	*C. parapsilosis*	>523	<0.25
7	11 days/F	VLBW (1255 g)	*C. parapsilosis*	>523	<0.25
8	120 days/M	LBW (1600 g)	*C. albicans*	190	1.37
9	120 days/M	LBW (1570 g)	*C. albicans*	230	1.05
10	145 days/M	LBW (1600 g)	*C. glabrata*	183	1.50
11	13 years/M	NHL	*C. albicans*	98	<0.25
12	11 years/M	ALL	*C. albicans*	100	1.15
13	5 years/F	AML	*C. parapsilosis*	310	<0.25
14	2 years/F	ALL	*C. parapsilosis*	222	<0.25
15	2 years/M	ALL	*C. lusitaniae*	135	0.71

BDG, (1→3) β-d-glucan; CM, *Candida* mannan; M, male; F, female; ELBW, Extremely Low Birth Weight (≤1000 g); VLBW, Very Low Birth Weight (1001–1500 g); LBW, Low Birth Weight (1501–2500 g); ALL, acute lymphoid leukemia; AML, acute myeloid leukaemia; NHL, non-Hodgkin’s lymphoma.
